# Circulating microRNAs in Cancer: A 5-Year Update with a Focus on Breast and Lung Cancers

**DOI:** 10.3390/ijms25063140

**Published:** 2024-03-08

**Authors:** Dario Siniscalco, Umberto Galderisi, Gianfranco Peluso, Mauro Finicelli

**Affiliations:** 1 Department of Experimental Medicine, Division of Molecular Biology, Biotechnology and Histology, University of Campania, 80138 Naples, Italy; dario.siniscalco@unicampania.it (D.S.); umberto.galderisi@unicampania.it (U.G.); 2 European Biomedical Research Institute of Salerno (EBRIS), 84125 Salerno, Italy; 3 Research Institute on Terrestrial Ecosystems (IRET), National Research Council of Italy (CNR), Via Pietro Castellino 111, 80131 Naples, Italy; gianfranco.peluso@unicamillus.org; 4 Faculty of Medicine and Surgery, Saint Camillus International University of Health Sciences, Via di Sant’Alessandro 8, 00131 Rome, Italy

**Keywords:** miRNAs, breast cancer, lung cancer, immunotherapy, recurrence

## Abstract

Circulating microRNAs (c-miRNAs) are non-coding RNAs found in different bodily fluids and are highly investigated for their prognostic potential and biological role in cancer. In this narrative review, we provide an update of the last five years’ published papers (2018–2023) on PubMed about c-miRNAs in cancer research. We aim to capture the latest research interests in terms of the highly studied cancers and the insights about c-miRNAs. Our analysis revealed that more than 150 papers focusing on c-miRNAs and cancer were published in the last five years. Among these, there was a high prevalence of papers on breast cancer (BC) and lung cancer (LC), which are estimated to be the most diagnosed cancers globally. Thus, we focus on the main evidence and research trends about c-miRNAs in BC and LC. We report evidence of the effectiveness of c-miRNAs in hot topics of cancer research, such as, early detection, therapeutic resistance, recurrence risk and novel detection platform approaches. Moreover, we look at the deregulated c-miRNAs shared among BC and LC papers, focusing on miR-21 and miR-145. Overall, these data clearly indicate that the role of c-miRNAs in cancer is still a hot topic for oncologic research and that blood is the most investigated matrix.

## 1. Introduction

Non-coding RNAs (ncRNAs) encompass a large family of RNAs not encoding for a known protein but having a role in the transcription of protein-coding genes, the maturation of the transcripts and their translation into functional proteins [1,2,3]. Among them, the micro RNAs (miRNAs) are the most studied and characterized type. The biological role of these molecules in both physiological and pathological processes has been widely investigated, as well as their potential applications for the diagnosis and prognosis of human diseases, including cancer [2].

MiRNAs are single-strand ncRNAs of approximately 22 nucleotides mainly involved in the post-transcriptional regulation of target mRNAs. They bind to the 3′-untraslated region of mRNAs and promote their degradation and/or inhibition of translation. The interaction of miRNAs with other regions, including the 5′ UTR, the coding sequence, and gene promoters, have also been reported. The interaction between miRNAs and target transcripts is dynamic and dependent on many factors, such as the subcellular location of miRNAs, the abundancy of miRNAs and target mRNAs, and the affinity of miRNA–mRNA interactions [4]. Given that a single miRNA can target up to 400 different target mRNAs, they are involved in several biological processes (proliferation, differentiation and apoptosis), as well as in the regulation of key genes, including oncogenes and tumor suppression genes. MiRNAs’ deregulation has been widely reported in many cancers [5,6]. Most of miRNAs act endogenously, but low levels of these molecules have also been found in the extracellular environment. MiRNAs have been found in cell culture media and in various biological fluids, such as blood (plasma and serum), saliva, urine, tears, semen, cerebral spinal fluids and ocular fluid; these molecules are referred to as circulating miRNAs (c-miRNAs) [7,8,9]. This was initially surprising, given the labile nature of RNA and the presence of RNases in biological fluids. Further studies demonstrated that the c-miRNAs in the extracellular space were associated with proper carriers preserving them from degradation and increasing their stability.

The functional role of c-miRNA seems to be intracellular communication, given that miRNA-dependent cell-to-cell communication is a key step for many physiological and pathological processes. For example, c-miRNA could be secreted by cancer cells triggering tumorigenesis in neighboring cells [10]. Besides the intracellular function of miRNAs, the alteration in the expression pattern of extracellular miRNAs is associated with the origin, progression and therapeutic response to cancer [11]. Indeed, c-miRNAs have been investigated as biomarkers to distinguish cancer patients from heathy subjects. Recently, many studies have suggested that c-miRNAs seemed to be involved in the regulation of inflammation and the epigenetic profile, as well as in predicting unhealthy incidents [10,12].

**Figure 1:**
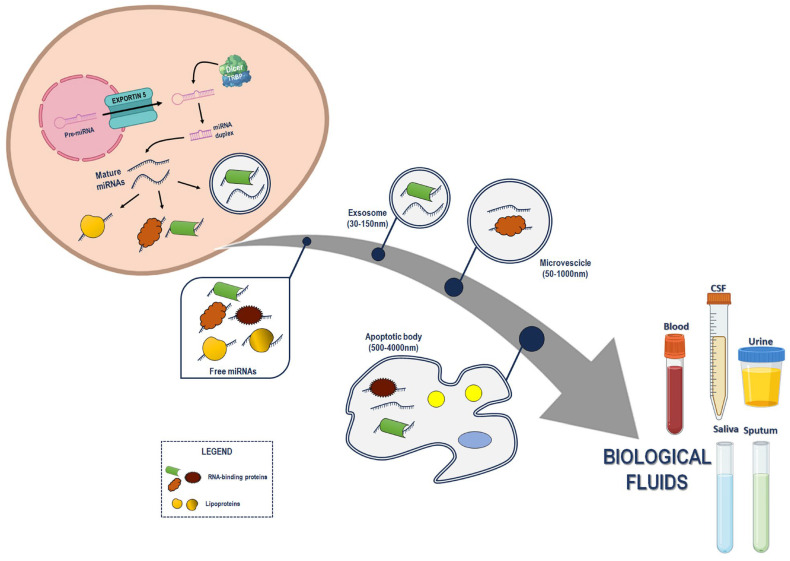
Schematic presentation of the miRNA’s cellular release into circulation. The pre-miRNAs are exported to the cytoplasm where they are processed by the Dicer/TRBP complex to produce the miRNA duplex intermediate. The interaction with Ago and the RNA-induced silencing complex (RISC) in the cytoplasm leads to the final miRNA maturation. Then, miRNA can be secreted from the cells by passive or active release as free circulating miRNA (complexed with RBPs or lipoproteins), or encapsulated in vesicles (i.e., exosomes, microvescicles or apoptotic bodies). CSF = cerebral spinal fluids. All the icons for biological fluids, cells and miRNAs were created with BioRender.com (accessed on 25 January 2024).

MiRNAs may be (i) encapsulated in extracellular vesicles (EVs), such as exosomes, microvesicles, and microparticles; (ii) associated with carrier proteins (such as nucleophosmin 1—NPM1, and Argonaute protein 2—Ago2); (iii) or complexed with lipoprotein (including high- and low- density-lipoproteins) [13] (Figure 1). Despite these advances regarding c-miRNAs, the evidence about their origin is still controversial. Passive or active secretions into the extracellular space are the two non-mutually exclusive mechanisms commonly proposed. The former hypothesis considers the passive release of miRNAs into extracellular space from broken cells resulting from injury, cells undergoing death (apoptosis or necrosis), cells exposed to chronic inflammation, or from cells with a short half-life (e.g., platelets). On the other hand, cells may actively secrete miRNAs as signaling molecules regulating cell-to-cell communications [6,14,15]. An example of the active transport of miRNA is mediated by EVs that are generated by the fusion of multivesicular bodies and the plasma membrane. Although the mechanism of release is accepted, the process determining the miRNA content in EVs is still under debate. Some studies have focused on the role of Ago2, given that this protein seemed to co-localize with the exosome protein CD63 in cytoplasm during the formation of multivesicular bodies. Moreover, Ago 2 phosphorylation has been proposed to be involved in key processes such as miRNA sorting and loading along with exosome release [16]. Another mechanism of miRNA release consists of the complex with carriers. As previously discussed, low-density and high-density lipoproteins, Ago-2 and NPM-1, can transport the miRNA in the circulation, preserving them from degradation [16].

The participation of miRNAs in many pathological processes, the presence of c-miRNAs in biological fluids, the high stability of c-miRNAs, the resistance of c-miRNAs to ribonucleases and physiochemical conditions are the principal properties increasing the attractiveness of these molecules as suitable biomarkers for cancer. Although the gold standard for cancer diagnosis is the histological analysis of tissue biopsies, the identification of alternative and less-invasive tools is still a need to corroborate the current screening programs aiming to better the monitorization of treatment response and disease progression [2,7]. In this context, liquid biopsies emerge as suitable analytic matrixes by providing a systemic panorama of the tumoral landscape [7]. The first evidence about the possible use of c-miRNAs as accessible biomarkers was posed in 2008 [17,18]. Through the years, the literature about the diagnostic and predictive role of c-miRNAs in cancer has increased exponentially, leading to the initiation of clinical trials, of tailored studies toward understanding their biological functions and of the standardization of the miRNA analysis field [6,19,20].

In this narrative review, we provide an update of the publications related to c-miRNAs in cancer research during the last five years, aiming to speculate the latest research interests in terms of the most studied cancers and the insights about c-miRNAs.

## 2. C-miRNAs in Cancer: An Update of the Past Five Years’ Literature

**Figure 2:**
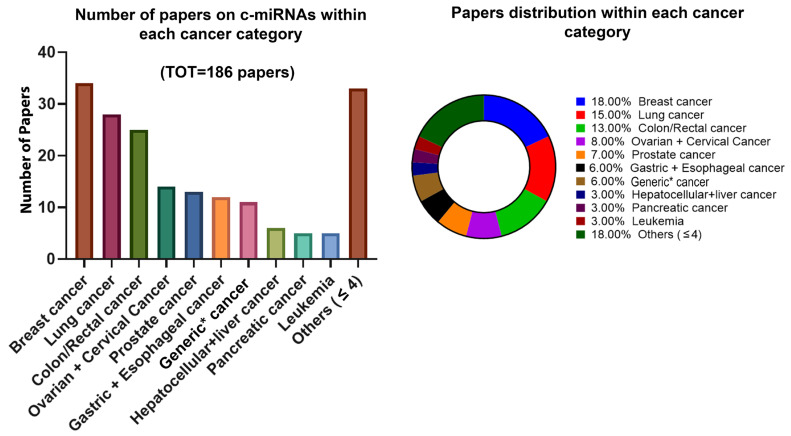
Charts showing the number (on the **left**) and the percentage (on the **right**) of publications related to c-miRNAs in cancer research in the last five years (2018–2023). Data from PubMed using search terms: “circulating microRNA cancer” and “circulating microRNAs cancer” (accessed on May 2023). * This group encompasses the papers in which two or more cancers were considered.

As previously discussed, the first evidence about the role of c-miRNAs in cancer was published in 2008 [17,18]. Since then, a huge number of publications about the applicability and the effectiveness of miRNAs as diagnostic and prognostic biomarkers in cancer have spread out. In May 2023, we provided an update of the last five years of published papers (2018–2023) on PubMed about circulating miRNAs in cancer research. We used the following search terms: “circulating microRNA cancer” and “circulating microRNAs cancer”, and the filters: “Title/Abstract” and “Human” were applied. The common papers between the two searches were detected and considered individually. The resulting papers were further revised so the review and the meta-analysis articles were censored, given we aimed to provide evidence only from original papers focused on c-miRNAs in cancer. These analyses allowed us to reveal that 186 papers were published on PubMed in the last five years, and we categorized them according to cancer type (Figure 2). The less frequent cancers (<four papers) were collected in the group “others”. Taken together, these data confirm the increasing interest in the research of c-miRNAs in cancer, as suitable biomarkers in a cancer diagnostic, prognostic, and treatment response. Looking at the categories listed in Figure 2, all the most prevalent types of cancers are present (i.e., breast cancer, lung cancer, colorectal cancer and gastric cancer). This led us to speculate the growing interest for c-miRNAs in oncological research, suggesting that the potential of these molecules needs to be further addressed. These data are in line with the evidence reporting the international effort in terms of the understanding of c-miRNA functions and the standardization of detection procedures [6].

The most studied cancers on our list were breast cancer (BC, 18%) and lung cancer (LC, 15%) (Figure 2). These data are in line with the recent epidemiology reports, which assess these cancers to be the most diagnosed worldwide [21,22]. Of note, the GLOBOCAN 2020 estimates of cancer incidence and mortality produced by the International Agency for Research on Cancer revealed BC to be the most commonly diagnosed cancer (11.7% of total cases) followed by LC accounting for 11.4% [21]. Looking at the mortality rate for each sex, BC is the deadliest cancer in females (15.5%), while LC is the deadliest in males (21.5%) [21]. Overall, these findings let us speculate that the identification of effective preventive and curative interventions is still a pressing problem for cancer. In particular, the role of c-miRNAs in breast and lung cancers could provide effective advances for cancer diagnosis, prognosis, and management. In the following paragraphs, we have provided a focus on the main evidence and research trends regarding c-miRNAs and BC and LC.

## 3. BC and C-miRNAs

BC is the most diagnosed and deadly cancer worldwide; 2.3 million cases of BCs were estimated in 2020. It is the most common cancer in women, with a high rate of incidence (24.5% of all cancer cases) [21,23]. Moreover, BC is the second leading cause of cancer-related death in more developed countries, after lung cancer [24].

To date, early detection is the gold standard for optimizing the management of BC and decreasing the death rate. Although mammography, ultrasound, MRI and invasive core needle biopsy are the current diagnostic tools, several limitations still exist, such as the exposure to X-ray radiation and/or a minimum tumor volume for detection [25,26]. Blood biomarkers are useful for monitoring BC treatment, but they lack sensitivity for primary cancer detection [25]. Translational research is still needed for the identification of novel robust prognostic markers and clinical targets for BC; circulating miRNAs could serve as such.

### 3.1. C-miRNAs and BC Diagnosis

Ali and colleagues evaluated the associations between the expression levels of serum miR-182 and -375 with BC and routine biochemical markers [27]. Their study group encompassed pre- and post-menopausal women suffering from BC, patients with benign tumors and healthy age-matched controls. Both miRNAs were highly expressed in pre- and post-menopausal BC patients as well as in those with benign tumors. Of note, miR-182 and -375 were positively associated with estrogen and progesterone receptors, suggesting roles as promising diagnostic and prognostic biomarkers for BC [27]. Analogously, another interesting study provided the prognostic potential of two other circulating miRNAs, i.e., miR-195 and -331 [28]. The authors identified a subset of circulating miRNAs by testing a discovery and validation cohort of BC patients affected by a local disease (Luminal A) and distant metastatic disease with respect to healthy controls. Among the dysregulated miRNAs, miR-331 and miR-195 resulted in being over- and under-expressed, respectively, in patients with metastatic BC compared to those suffering from a local disease or those who were heathy subjects. The combination of these expression profiles provided a robust statistical result (Area Under the Curve − AUC = 0.902), demonstrating the effectiveness of circulating miRNAs in identifying metastatic disease [28]. The combinatory analysis of serum and plasma samples from BC patients and healthy controls (HCs) was further carried out to dissect the prognostic value of 12 miRNAs belonging to the miR-106a-363 cluster, located on human chromosome X and scarcely analyzed in BC despite the gender incidence of this malignancy. Four plasma miRNAs (miR-106a-3p, miR-106a-5p, miR-20b-5p, and miR-92a-2-5p) and four serum miRNAs (miR-106a-5p, miR-19b-3p, miR-20b-5p, and miR-92a-3p) were upregulated in BC, identified, and their prognostic significance was demonstrated. Of note, the two overlapping miRNAs, miR-106a-5p and miR-20b-5p, were overexpressed in BC tissue too, suggesting a potential role in cancer biology [26].

Zou and colleagues found a 12-miRNAs panel, whose serum level was upregulated in BC patients with respect to HCs. Their analyses revealed that this panel (let-7b-5p, miR-106a-5p, miR-19a-3p, miR-19b-3p, miR-20a-5p, miR-223-3p, miR-25-3p, miR-425-5p, miR-451a, miR-92a-3p, miR-93-5p, and miR-16-5p) was able to distinguish BC in different stages from controls [29].

Overall, these results confirm the high prognostic potential of circulating miRNAs in BC.

The research has also focused on the miRNAs composing the cargo of circulating EVs. Ozawa and colleagues carried out an intriguing study, in which the cargo of EVs derived for the sera of CT subjects (healthy controls), LAs (Luminal A BC patients) and TNBCs (triple negative BC patients), was analyzed using RNA-seq theology [30]. Their data showed the differential expression profiles of four EV-miRNAs, i.e., miR-142-5p, miR-150-5p, miR-320a, and miR-4433b-5p, among the groups. The quantitative real-time PCR analysis on a validation cohort revealed that a panel of miR-142-5p, miR-320a, and miR-4433b-5p was able to distinguish BC patients from CT subjects. Moreover, the combination of miR-142-5p and miR-320a was associated with LAs with respect to CT subjects (AUC of 0.9410) and the decrease in the expression levels of miR-142-5p and miR-150-5p was related to advanced-grade BC (grade III). These data suggested the predictive effectiveness of circulating EV-miRNAs [30].

### 3.2. C-miRNAs and Genetic and Environmental Factors in BC

An interesting aspect emerging from the selected papers is the impact of genetic and environmental factors that could influence BC malignancy. Some studies focused on the differential expression of circulating miRNAs in different ethnic groups. For example, miR-202 was overexpressed in BC patients from South Korea, and the expression levels of miR-21, miR-155, miR-23a, miR-130a, miR-145, miR-425-5p, and miR-139-5p were upregulated in Lebanese BC patients [31,32]. No overlap arose from these studies, suggesting a possible involvement of genetic or environmental factors. Indeed, Uyisenga and colleagues tested a previously identified miRNA signature in two different populations, from Belgium and Rwanda. Despite the circulating miRNA signature showing diagnostic significance in each population, it lacked effectiveness when both the populations were considered as a unique cohort [25]. On the other hand, Zou and colleagues provided an intriguing multicenter study, involving both Caucasians and Asians, testing the prognostic value of circulating miRNAs. Interestingly, they found an eight-miRNA signature, consisting of four upregulated (miR-133a-3p, miR-497-5p, mir-24-3p, and miR-125b-5p) and four downregulated (miR-377-3p, miR-374c-5p, miR-324-5p and miR-19b-3p) miRNAs, able to distinguish between BC patients and healthy individuals [33]. Overall, these findings led us to speculate that controversies still remain in this field and further efforts must be provided to improve our knowledge.

### 3.3. C-miRNAs and BC Treatments

Another important clinical aspect for BC is the treatment efficacy and management, in order to avoid adverse effects or not-responding issues. The recent literature on circulating miRNAs also focused on this feature. Of interest, Anwar and colleagues looked at the clinical significance of circulating miR-155 expression profiles in a cohort of 105 BC patients at diagnosis and after treatment. Their data showed an increase in circulating miR-155 in BC patients rather than in HCs (n = 15). The interesting evidence was that the miRNA levels decreased following surgery and chemotherapy, suggesting a potential role of miR-155 in diagnosis and therapeutic monitoring [24]. Analogously, Ibrahim and colleagues investigated the expression levels of a panel of oncomiRs and tumor suppressor miRNAs in a cohort of locally advanced BC patients at diagnosis, during treatment, and after tumor restriction. They found a differential expression of several miRNAs in BC patients with respect to HCs; miR-21, miR-181a, and miR-10b were upregulated, whereas miR-145 and let-7a significantly decreased. Interestingly, the expression levels of these putative biomarkers returned to control values once the treatment finished. Moreover, miR-10b and miR-21 showed a predictive significance in detecting progression-free survival [34]. The prognostic efficacy of other oncomiRs and tumor suppressor miRNAs were analyzed in the plasma of non-metastatic Luminal A patients undergoing common treatments, such as surgery, chemotherapy, and radiotherapy. Quantitative analyses revealed that the circulating levels of miR-21, miR-55, and miR-10b were upregulated in BC patients compared with healthy controls with the concomitant decrease of the tumor suppressor miRNA let-7a. Interestingly, the treatments reversed the expression patterns of both oncomiRs and tumor suppressor-miRNAs [35]. These findings open an intriguing scenario, given that the ability of understanding the response to treatment could be extremally useful in avoiding some issues, such as side effects, under- or over-treatments.

Neoadjuvant chemotherapy (NAC) has shown high potential for early and local-advanced BC treatment, aiming to improve the pathologic complete response (pCR) and consequentially to increase patient survival. Thus, the prediction of a patient’s response to NAC has a high significance in terms of avoiding side effects and lowering ineffective treatment. In this scenario, the recent literature has pointed out the associations between circulating miRNAs and the response to NAC, looking at their predictive potential. Davey and colleagues demonstrated that the increase of Let-7a and the decrease of miR-145 predicted the response to NAC in Luminal B and HER2+ BCs, respectively [36]. Moreover, in an Irish multicenter translational research trial, the increased expression of circulating miR-145, detected in the midway during NAC, was associated with improved recurrence-free survival in the study population [37]. The circulating levels of miR-718, miR-4516, miR-210, and miR-125b-5p were associated with chemosensitivity in Luminal B-HER2-negative patients undergoing NAC [38]. Analogously, the dynamics of three miRNAs (i.e., miR-222, miR-20a, and mir-451) were associated with chemosensitivity in a cohort of HR+/HER2+ BC patients [39].

The prognostic value of circulating miRNAs was also demonstrated in HER2-positive BC patients receiving trastuzumab-based neoadjuvant therapy, belonging to the NeoALTTO trial. The comparison of plasma pairs collected prior to and two weeks after treatment revealed an increase in the levels of miR-148a-3p and miR-374a-5p that correlate with pCR. Of note, the early modulation of miR-148-3p seems to bestow a high prognostic significance in identifying patients likely to respond to therapy and be implicated in the functional pathways associated with treatment response [40].

### 3.4. C-miRNAs and BC Recurrence

Other key aspects characterizing the malignancy of BC are the local or distant recurrence, which affect 30% of BC patients. The identification of biomarkers able to better predict the risk of recurrence as well as the management of BC patients is still a huge need [41,42]. Even in this case, the circulating miRNAs promise high potentiality. Fisher and colleagues studied the correlations among the expression levels of the plasma miR-200 family and some BC features and outcomes, such as the survival rate, circulating tumor cell count, and response to treatment. MiR-200a, miR-200b, miR-200c, miR-141, and miR-429 belong to the miR-200 family and are mainly involved in the epithelial-to-mesenchymal transition, an essential process in the metastatic cascade. The BC patients receiving a complete cycle of systemic therapy showed a reduction in the expression levels of miR-200a, miR-200b, and miR-141. These values returned to the basal level upon the progression of disease. Interestingly, the expression levels of the miR-200 family were upregulated in the BC patients showing circulating tumor cells. These data suggest the potential effectiveness of these miRNAs in the management of metastatic BC [43,44]. Analogously, Papadaki et al. investigated the prognostic efficacy of a five-miRNA panel in the plasma of BC patients before adjuvant chemotherapy. Their data showed that patients suffering a relapse had higher levels of miR-21, miR-23b, and miR-200c with a concomitant decrease in mir-190 with respect to non-relapse patients. Moreover, an miR-190 reduction was observed in patients with an early relapse (<3 years) while increased levels of mir-21 and miR-200c were associated with a late relapse (>5 years). The combination of the miR-200c expression levels with other clinicopathological parameters, such as lymph node infiltration, tumor grade, and estrogen receptor status, predicted late relapse in this BC cohort, suggesting their intriguing efficacy as biomarkers for BC recurrence [41].

Finally, an analysis of the plasma levels of miR-199a and miR-633b was carried out in a small cohort of metastatic breast cancer patients and healthy subjects to investigate their association with chemotherapy response. Interestingly, high levels of miR-199a and low levels of miR-633b significantly correlated with chemoresistance in BC metastatic subjects [45]. Despite the limitation of the study, this is a further indication of the predictive effectiveness of the circulating miRNA in BC diagnosis and management.

### 3.5. Novel Detection Systems and Approaches for C-miRNA Detection in BC

Another key topic attracting the research on circulating miRNAs is the development of rapid, sensitive, and effective detection platforms able to improve those commonly used. Although the northern blot, reverse transcription quantitative polymerase chain reaction (qRT-PCR), next-generation sequencing, and microarray are the widest and most robustly used methods by researchers, the problem of specificity still exists [46,47,48]. Some strategies are aimed at a reasonable combination and improvement of the common approaches. For example, Li and colleagues provided a highly sensitive graphene oxide (GO)-based qRT-PCR for circulating miRNA detection. They hypothesize that GO could increase the annealing temperature of the primer template and limit the inhibitory effect of other substances to the process, thus improving the efficiency and sensitivity of qRT-PCR. Moreover, the authors used this method to assess the expression levels of three circulating miRNAs, such as miR-21, miR-155, and miR-195, in a BC cohort treated with NAC. They showed that miRNA levels seemed to distinguish the NAC-responder patients from those non-responders [49]. Another method carried out to improve the standard qRT-PCR technique for miRNA quantitative analysis is the branched rolling circle amplification (BRCA). This is an exponential nucleic acid amplification method based on an isothermal enzymatic one-step reaction with a further amplification by the addition of a second and third primer. For the first time, Fan et al. demonstrated the detection of circulating miRNAs using a one-step BRCA of serum samples from BC patients. They demonstrated that the BRCA approach detected the differences in the expression levels of miR-155 and miR-195 in BC patients vs. healthy subjects. These results were further validated with the conventional qRT-PCR method and the diagnostic accuracy of miRNA expression levels for BC early diagnosis was confirmed using a receiver operating characteristic (ROC) curve assay [50].

Nanotechnology-based approaches have been further investigated as alternative strategies to the conventional methods. A label- and enzyme-free optical copper nanocluster-DNA-based biosensor was developed for multiple detections of circulating microRNAs in BC patients. This method was properly adjusted to allow for the simultaneous detection of three oncomiRs (miR-21, miR-195, and miR-155), with a significantly low LOD (1.7 pM) within the target molecule concentrations range of 500 nM to 3 μM [51]. Analogously, a colorimetric biosensor obtained using a split G-quadruplex (Gq) nanostructure and its peroxidase-mimicking property was carried out to detect miRNAs in blood. This method was able to distinguish target sequences from non-target ones in both buffer and blood media. Moreover, different concentrations of target sequences allowed researchers to set the detection limit to 0.38 nM with a linear response range from 0 to 10 nM [52]. Concerning the nano-based biosensor, an intriguing solution of miRNA detection was provided by Garrido-Cano and colleagues. They developed a nanoporous anodic alumina (NAA) biosensor able to detect plasma levels of miR-99-5p. This approach consisted in a NAA containing a fluorescent dye capping with a target-specific oligonucleotide. Once the latter recognized the miR-994-5p sequence (i.e., the target), the cap was displaced and the dye was released. The authors used this method to distinguish heathy controls from BC patients according to their miR-994-5p expression profile with high efficacy and sensitivity [53].

Finally, also, surface-enhanced Raman spectroscopy (SERS) and a surface plasmon resonance imaging biosensor have been investigated as innovative approaches for the detection of circulating miRNAs. They both were able to detect miRNAs in biological fluid, even if the SERS-based approach was tested in a BC mouse model [54,55].

Table 1 lists the main evidence regarding c-miRNAs and BC.

## 4. LC and C-miRNAs

LC is the most prevalent cause of cancer-related mortality and the non-small cell LC (NSCLC) is the widest form, accounting for 85% of lung cancer patients. This form of LC is often diagnosed late and once the advanced state occurs, the therapeutic options are scarce [56,57]. The identification of circulant biomarkers able to identify LC in early stages, especially NSCLC, is still a pressing need to improve the prognosis of such an aggressive cancer.

### 4.1. C-miRNAs and LC Diagnosis

Khandelwal and colleagues provide a combinatory approach to assess the prognostic and functional role of miR-590-5p in NSCLC [58]. The authors firstly proved the significant downregulation of miR-590-5p expression levels in a cohort of 80 NSCLC patients compared to healthy controls. They also showed an association between low levels of miR-590-5p and a poor prognosis, in terms of median survival. Further, in vitro analysis of the human lung cancer cell line revealed that miR-590-5p targeted STAT3, negatively regulating its activation and its downstream signaling molecules involved in tumorigenesis, such as Cyclin D1, c-Myc, Vimentin, and β-catenin. Overall, these data led us to hypothesize that miR-590-5p may act as a tumor suppressor in NSCLC via the STAT3 pathway, and could be a useful biomarker for the diagnosis/prognosis of NSCLC [58].

The expression levels of six miRNAs were assessed in peripheral blood samples from histopathologically diagnosed lung adenocarcinoma (AC; n = 30) and squamous cell carcinoma (SCC; n = 30) cases, compared with those from healthy individuals (n = 20) [59]. The data revealed an increase in the expression levels of mir-2114 and mir-449c in AC and mir-2115 in SCC. The upregulation of mir-2114 and mir-449c was also confirmed in both the 293T and A549 AC cell lines [59]. Although these data need to be challenged in a wider cohort of patients and associated with other clinicopathological parameters, this is the first evidence of the potential diagnostic significance of these miRNAs.

Fehlmann and colleagues investigated the use of c-miRNAs for LC detection in a multicenter cohort study [60]. Their retrospective cohort included 3046 blood samples from patients suffering from lung cancer (n = 606; both NSCLC and small cell lung cancer—SCLC), patients with non-tumor lung diseases (n = 593; mainly chronic obstructive pulmonary disease—COPD), patients with no pulmonary diseases (n = 883), and unaffected control participants (n = 964). The authors investigated the miRNA signature of the following three classification scenarios: First, they looked at distinguishing between LC-diagnosed subjects and all other individuals. A signature of 15 miRNAs was identified: miR-1285-3p; miR-205-5p; miR-1260a; miR-1260b; miR-3152-3p; miR-378b; miR-17-3p; miR-1202; miR-139-5p; miR-16-2-3p; miR-18a-3p; miR-23b-3p; miR-3907; miR-551b-3p; and miR-93-3p. When applied to the validation set, the accuracy, sensitivity, and specificity were 91.4% (95% CI, 91.0–91.9%), 82.8% (95% CI, 81.5–84.1%), and 93.5% (95% CI, 93.2–93.8%), respectively. Analogously, a 14-miRNA signature was used to discriminate between LC and non-tumor lung diseases: let-7g-3p; miR-1202; miR-1285-3p; miR-17-3p; miR-193a-5p; miR-205-5p; miR-21-3p; miR-3610; miR-4282; miR-4286; miR-452-3p; miR-516a-3p; miR-572; and miR-625-5p. Even in this case, the analyses revealed an accuracy of 92.5% (95% CI, 92.1–92.9%), a sensitivity of 96.4% (95% CI, 95.9–96.9%), and a specificity of 88.6% (95% CI, 88.1–89.2%). The last scenario involved early stage LC patients vs. individuals without LC, and a nine-miRNA signature was obtained: miR-1260a; miR-1260b; miR-1285-3p; miR-17-3p; miR-205-5p; miR-3152-3p; miR-374b-5p; miR-378b; miR564. The validation set showed an accuracy, sensitivity, and specificity of 95.9% (95% CI, 95.7–96.2%), 76.3% (95% CI, 74.5–78.0%), and 97.5% (95% CI, 97.2–97.7%), respectively. Consistent with these results, Abdollahi et al. looked at the diagnostic relevance of a panel of circulating miRNAs for NSCLC detection [61]. They tested the circulatory levels of four miRNAs, i.e., miR-21, miR-638, miR148, and miR-152, in 43 NSCLC patients and in non-cancerous subjects. Their data revealed that miR-21 increased in NSCLC patients compared to controls along with a concomitant downregulation of miR-638, miR-148a-3p, and miR-152-3p circulating levels. When the authors looked at the sensitivity of these miRNAs, they found different values ranging from 90% (miR-21-5p) to 68% (miR-638). Interestingly, the combinatory use of them showed an increase in sensitivity (96.4%) and specificity (86.67%), suggesting a strengthening in terms of diagnostic efficacy [61]. A five-miRNA signature able to detect NSCLS was also provided by Ying and colleagues [62]. They identified 35 candidate miRNAs using an accurate experimental workflow, by means of an analytically validated qPCR method, in multiple patient cohorts, independent from their smoking status, gender, and ethnicity. The authors recognized a signature of five miRNAs, consisting in the reduced expression of let-7a-5p and miR375 along with the concomitant increase of miR-1-3p, miR-1291, and miR-241-3p levels. This signature was further tested in a discovery and validation cohort. These analyses showed the higher sensitivity of this signature for early stage NSCLC (83.0% for stage I and the 81.3% for all stages), suggesting a non-invasive approach for detecting early stage NSCLC [62].

Zhang et al. provided a large cohort analysis for identifying serum miRNAs’ prognostic significance in advanced NSCLC [63]. Their data showed a five-miRNA signature (miR-191, miR-28-3p, miR-145, miR-328, and miR-18a) that was associated with three-year overall survival (OS) in both discovery (n = 345) and validation (n = 177) cohorts. Among them, miR-191 showed the highest association with three-year survival. This is of interest given the recent findings demonstrating the upregulation of this miRNA in cancerous vs. non-cancerous tissues and its role in sustaining the proliferation and migration of LC cells in hypoxic conditions [63,64]. Moreover, the authors found that the target genes regulated by the five miRNAs were mainly involved in inflammatory and immune response signaling [63].

### 4.2. C-miRNAs, LC and EVs

Looking at the papers from our update, the prognostic value of EV-derived miRNAs was also investigated in LC. As previously reported, c-miRNAs could be directly released into blood circulation or secreted as EV cargo. Vadla et al. carried out an interesting study addressing the effectiveness of plasma EVs and c-miRNAs as suitable biomarkers for the screening and treatment decision of NSCLC [65]. Firstly, the authors looked at the analysis of c-miRNAs as a minimally invasive approach to improve cancer diagnosis, given the high false-positive rate in NSCLC screening by low-dose computed tomography. To this purpose, plasma EVs and c-miRNAs were assayed in both NSCLC patients and high-risk subjects. This analysis showed a set of miRNAs, let-7-5p, miR-184 from EVs, and miR-22-3p from c-miRNAs, that was able to discriminate between the two groups. The authors also investigated the functional role of these miRNAs in LC biology, demonstrating their involvement in the WNT/βcatenin and mTOR/AKT signaling axes. Given the impaired regulation of these pathways is strictly related to therapy resistance in different types of cancer, authors have speculated the possible prognostic significance of these deregulated miRNAs for patient clinical outcomes. By means of an in vitro approach, the miR-184 and miR-22-3p targeting resulted in a desensitization of EGFR-mutated NSCLC cells to Osimertinib, a potent tyrosine kinase inhibitor commonly used in EGFR-mutated patients. These findings let authors speculate the effectiveness of the miRNA signature in helping clinicians form treatment decisions [65].

Concerned for the prognostic effectiveness of EV-miRNAs, Zhang et al. investigated the expression levels of serum exosomal miR-378 in NSCLC patients (n = 103), in subjects with a non-malignant disease (n = 32) and in healthy controls (n = 60) [66]. Their data showed that miR-378 was upregulated in cancerous sera compared with heathy sera, and this increase was also associated with lymph node metastasis and the TNM stage. Of note, radiotherapy-treated patients showed a decrease in serum exosomal levels of miR-378, suggesting an intriguing prognostic indication for therapy response [66].

### 4.3. C-miRNAs and LC Treatments

The effectiveness of circulating miRNAs as biomarkers has also been addressed for discriminating immunotherapy efficacy. Anti-programmed cell death protein 1 (PD-1) or programmed death-ligand 1 (PD-L1) antibody therapy is commonly used for advanced NSCLC. To date, a high immunohistochemical score for PD-L1 is a predictive marker of therapeutic response, although for some patients, it failed to be of prognostic value [67,68]. In this scenario, the circulating and EV-miRNAs are good sources of suitable biomarkers. Shukuya et al. used a next-generation sequencing platform to analyze the miRNA profile of both whole plasma and plasma EVs from a small cohort of advanced NSCLC patients [69]. All the subjects were treated with a single-agent anti-PD-1 or an anti-PD-L1 antibody. They were divided into two groups, responders and non-responders, according to the exhibition of a partial response or stable disease (>6 months) and of a progressive disease, respectively. The expression patterns of 32 c-miRNAs and 7 EV-associated miRNAs differed between the responder and non-responder patients. Among the 32 c-miRNAs, the authors identified miR-199a-3p, miR-200c-3p, miR-21-5p, miR-28-5p, and miR-30e-3p, whose expression levels were further validated by qPCR. miRNA targets and gene enrichment analyses suggested that these miRNAs played a role in the cancer and immune system-related pathways. Further analysis allowed the authors to restrict the set to miR-200c-3p, miR-21-5p, miR-28-5p, whose expression levels significantly decreased in responders when compared to non-responders. These data allowed the authors to speculate that specific c-miRNA signatures could have potential as predictive biomarkers for anti-PD-1/PD-L1 treatment response [69]. Analogously, Fan et al. investigated the association between c-miRNAs and immunotherapy resistance in NSCLC patients [70]. The authors analyzed the sera of 19 responder and 27 non-responder patients, identifying 27 miRNAs whose expression results were dysregulated between the groups. Among them, ten miRNAs were highly expressed in an independent cohort of 17 responder patients compared with 17 non-responder patients: miR-93, miR-138-5p, miR-200, miR-27a, miR-424, miR-34a, miR-28, miR-106b, miR-193a-3p, and miR-181a. The statistic associations revealed that responders showed a 10-fold increase in the expression levels of these miRNAs from pre- to post-treatment. The highly expressed signature was further associated with the improvement of progression-free survival. The OS analysis in responder and non-responder patients also showed a prognostic significance of the upregulated miRNAs [70]. A systemic seven-miRNA signature (miR-215-5p, miR-411-3p, miR-493-5p, miR-494-3p, miR-495-3p, miR-548j-5p, and miR-93-3p) was further identified in NSCLC patients and was associated with OS after treatment with the immune check point inhibitor nivolumab. This finding was also confirmed in a validation cohort showing a sensitivity of 70% and a specificity of 90% [71].

Monastirioti et al. focused on the immunomodulatory potential of miRNAs, looking at the prognostic significance of those acknowledged for being involved in macrophage polarization [72]. Among them, high plasma levels of miR-202 correlated with disease progression in NCSLC patients and showed a prognostic significance for shorter progression-free survival and OS. Moreover, high miR-26a was associated with short OS in the squamous subgroup of the NCSLC patients [72].

### 4.4. Novel Detection Systems and Approaches for C-miRNA Detection in LC

Although most of the studies we found were carried out to test the effective diagnostic and/or prognostic effectiveness of c-miRNAs in LC, some studies addressed technical issues too, such as the setup of methods for a sensitive detection of these molecules in biological fluids. Meng and colleagues showed a versatile electrochemical biosensor for the detection of c-miRNAs [73]. This method consisted in a hybridization chain reaction (HCR) driven by a DNA walker combined with a glutathione-derived metal ion for determination. The HCR is an innovative amplification technique driven by a cascade of DNA polymerization by initiator or target molecules [74,75]. The authors demonstrated the effectiveness of this biosensing method, which showed a high selectivity in distinguishing between target and single-base mismatched miRNAs. This strategy was also tested by profiling NSCLC patients and demonstrated high efficiency in the identification of stage I cancers. Moreover, its ability in discriminating between NSCLC and benign disease was also proven, confirming the high promise of this application [73].

Tian et al. developed a novel nanoprobe approach to detect the serum level of miR-150 [76]. The authors set up a spherical nanoprobe labeled with a fluorophore and a quencher. These molecules formed a harping structure, through DNA self-assembly, that hid the signal when the target miRNA was absent. Once miR-150 was present, the target-probe hybridization moved the quencher away from the fluorophore, allowing for signal emission. The authors demonstrated the high sensitivity of the probe with a detection limit of 38fM. They also tested this method for assaying the expression of the serum levels of miR-150 in a very small cohort of NSCLC patients and healthy people, demonstrating the diagnostic effectiveness of the probe [76].

Among the new strategies for an accurate detection of miRNAs in biological fluids, those based on the rolling circle amplification (RCA) process are emerging. Both luciferase-DNA chimeras integrated into RCA and the photonic crystals-assisted RCA biochip showed promising potential in miRNA detection with an appreciable accuracy and sensitivity [77,78]. In both papers, these strategies offered novel potential tools for the implementation of miRNA identification in biological fluids.

An interesting novel approach has been provided by Detassis and colleagues. They showed a chemical-based method for accurate c-miRNA profiling from biologic fluids without preparatory steps (i.e., extraction, pre-amplification, or pre-labeling) [79]. Their approach consisted in a chemiluminescent reaction generating a signal which, in turn, was read by a novel silicone photomultiplier-based reader. This method was structured for has-miR-21-5p detection and showed a detection limit of 4.7 pmol/L. The authors also tested the platform for has-miR-21-5p detection in the plasma of eight NSCLC patients and compared the results with those arising from a TaqMan RT-qPCR assay. The promising evidence poses the basis for the development of the direct and quantitative analysis of miRNAs in biological fluids [79].

Table 2 lists the main evidence regarding c-miRNAs and LC.

## 5. The C-miRNAs Shared among the Selected Studies

**Figure 3:**
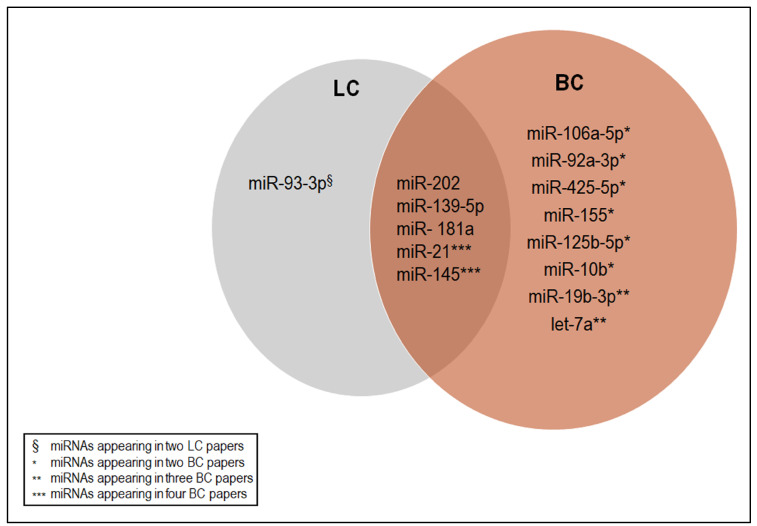
Venn diagram showing the deregulated c-miRNAs shared among the studies cited in the present review.

Looking at the dysregulated c-miRNAs in BC and LC, we identified those overlapping among the studies reported in the present review. This would be useful for pointing out the most representative c-miRNAs among the selected studies. The indications provided could be useful for setting out tailored studies, as well as the initiation of clinical trials to monitor cancer diagnostics and/or monitoring the response to therapies. The identification of common c-miRNAs in BC and LC could provide further insights into the promising role of these molecules in oncology and clinic research. As shown in Figure 3, BC papers had most of the shared miRNAs, ranging from the low representative (i.e., miRNAs appearing in two papers, at least, even in a signature) to the highly representative (i.e., miRNAs appearing in four papers, even in a signature).

When we looked at the shared c-miRNAs whose expression was dysregulated in both BC and LC, we found five: miR-202, miR-139-5p, miR-181a, miR-21, and miR-145 (Figure 3). Among them, we focused on miR-21 and miR-145, which were the highly representative miRNAs in BC that were also present in LC. Both the miRNAs were cited in five papers (4 BC + 1LC), confirming the interesting role of these molecules in cancer.

Cancer patients showed high levels of miR-21 in all five studies [31,34,35,41,61]. The concordance in the detection procedures (qRT-PCR) and sampling (blood) contributed to increasing our interest in miR-21. Although the expression level of this miRNA was included in some signatures, its upregulation seems to have a diagnostic significance in both early-stage BC and NSCLC detection, as well as a prognostic role in detecting BC-relapsed patients. These findings are in line with the evidence about the efficacy of serum miR-21 in the early detection of BC and NSCLC [41,61,80,81]. Moreover, concordance exists in associating the expression levels of miR-21 with a poor prognosis in BC, in terms of distant metastatic risk and of its ability in predicting the response to chemotherapy [82,83,84]. The oncogenic role of miR-21 in BC and LC seemed to corroborate this evidence. In BC, miR-21’s tumor-promoting effects could be exerted by the regulation of transforming growth factor-beta (TGFβ) signaling, given that both TGFβ1 and its receptor TGFβR2 are putative targets of this miRNA. Other studies revealed that miR-21 overexpression induced the suppression of the TGFβ/Phosphatase and tensin homolog (PTEN) axis contributing to BC progression and chemoresistance [85]. The miR-21-mediated PTEN suppression was also demonstrated in NSCLC patients and was associated with a resistance to target therapy [86]. Moreover, miR-21 seems to be involved in other cancerous processes (i.e., cell invasion, metastasis, and angiogenesis) via its targeting of key mRNA players, such as the tissue inhibitor of matrix metalloproteinases 3 (TIMP3), the programmed cell death receptor 4 (PDCD4), tropomyosin 1 (TPM1), and the reversion-inducing cysteine-rich protein with kazal motifs (RECK).

The expression pattern of miR-145 is controversial in the five papers reporting its systemic dysregulation in cancer [31,34,36,37,63]. The upregulation and downregulation of this miRNA were reported in BC papers. Dovey et al. detected low levels of miR-145 in patients who achieved a pCR to NAC in HER2+ (ER−/HER2+) disease, demonstrating a concordance with the results of the translational study of the NeoALTTO trial [36]. On the other hand, the increase in the expression levels of miR-145 was reported in Lebanese and Irish BC patients, although its downregulation in tissue and plasma samples from different ethnic groups has been also demonstrated [31,37]. A tumor suppressor role for miR-145 in BC has been hypothesized, given its inhibitory effect on cell migration and its suppression of the epithelial-to-mesenchymal transition [87]. An increase in serum levels of miR-145 was observed in LC and it was associated with poor survival in patients with advanced NSCLC [63]. As in BC, miR-145 seemed to inhibit the migration and invasion of lung cancer cells by targeting the gene FSCN1 [88].

Overall, these findings suggest that the evaluation of dysregulated miRNAs in larger experimental groups, particularly miR-145, is necessary. This is due to the fact that miR-145 may play different roles in tumor cells and in circulation [63].

Taking a brief look to the other three miRNAs, miR-202 expression levels have been seen to be upregulated in the blood and serum of BC patients. However, a differential and variable involvement of this miRNA in tumorigenesis has been hypothesized, as decreased levels of miR-202 have been found in the tumor tissues of different types of cancer. MiR-202’s involvement in signaling pathways foretells the pro- or anti-tumorigenic response of its dysregulation and eventual biomarker capacity [89].

Increased levels of circulating miR-202 were found in BC patients in respect to healthy controls, suggesting its potential role as diagnostic tool for detecting BC in its early stages [32].

It has been hypothesized that miR-181a is able to downregulate the stimulator of interferon genes (STING) and pro-inflammatory cytokines to mediate PARP inhibitor drug resistance [90].

Indeed, in BC, the pathway NF-κB/IL-6/STAT3 is responsible for miR-181a upregulation, promoting chemotherapy resistance and inducing metastasis [91].

Conversely, the expression levels of miR-181a-5p have been found to be decreased in BC tissues and cell lines. MiR-181a-5p targets the mRNA 3′-UTR region of the tumor suppressor genes KLF6 and KLF15, and its overexpression decreases cell proliferation [92].

It has been proposed that circulating miR-181a-5p could serve as a non-invasive biomarker for non-small cell LC. Indeed, tissue and plasma expression levels decreased in non-small cell LC and the rates of the progression-free survival of the patients were longer [93]. Furthermore, it was found to be downregulated in early-stage LC; its role in tumor initiation and progression indicates its value as an early-stage biomarker and treatment target [94].

MiR-139-5p is a cancer suppressor, capable of inhibiting cancer cell proliferation and enhancing apoptosis [95,96]. Its plasma levels have been proposed to be non-invasive biomarkers for LC diagnosis [97].

Interestingly, it has been demonstrated that human bone marrow mesenchymal stem cells are able to inhibit BC cells’ growth through the release of exosome cargo with miR-139-5p [98].

MiR-139-5p has been included in a panel of an 8-miRNA signature predictive model to provide a prognosis for triple-negative BC patients with a high risk of relapse after surgery [99].

## 6. Conclusions

The analysis of the existing literature on PubMed revealed a significant increase in publications focusing on the role of c-miRNAs in cancer, with >150 papers in the last five years. Among these, there was a high prevalence of papers on BC and LC, which have been estimated as the most diagnosed cancers globally.

Overall, these data clearly indicate that the role of c-miRNAs in cancer is still a hot topic for research. The recent body of literature on c-miRNAs pairs with the main topics of cancer, such as therapeutic resistance, the need of more sensitive diagnostic procedures and improvement in patients’ management.

It is interesting to point out that blood is the most investigated matrix for c-miRNAs’ expression in all the papers selected in our update. The impact of genetic and environmental factors influencing BC has emerged as an intriguing aspect that needs to be properly investigated, given it could affect subject enrollment in clinical trials. The controversies on the effectiveness of c-miRNAs for different ethnic groups still exist and further studies must be carried out.

Finally, the development of novel methods and approaches for c-miRNAs’ detection, analysis, and quantification has received much attention in both LC and BC research. Nanostructure-based assays, innovative biosensors, and effective detection platforms have been proposed as novel and more sensitive systems for a more accurate and standardizable identification of miRNAs in biological fluids.

An interesting speculation is the focus on the highly representative miRNAs in the studies reported in the present review. This led us to identify miR-21 and miR-145 as promising candidates for being suitable biomarkers for BC and LC, although their effectiveness must be confirmed in large experimental studies.

Despite the promising advances in this field, further efforts are needed to corroborate the intriguing findings obtained. Prospective studies with tailored and large cohorts of patients will provide insights into the extent to which miRNAs complement imaging and biopsies in clinical application. The lack of standardization in terms of pre-analytical and analytical workflow and the use of multiple quantification strategies are still strong limitations affecting the concordance among the existing studies. The identification of robust biomarkers needs to be established in guidelines useful for testing and validating these candidates in prospective clinical studies.

Moreover, an in depth focus on the biological functions of the identified c-miRNAs could be also useful in determining their prognostic effectiveness, as well as providing more indications about their systemic response to cancer. An improved knowledge of the mechanisms regulating miRNA secretion and transport, as well as the understanding of the effects in upstream and/or downstream pathways are still a pressing need. Another important question that needs to be addressed regards the main features of c-miRNAs in regulating cell-to-cell communication [10]. These insights could contribute to overcoming the barriers outstanding the clinical use of c-miRNAs.

## Figures and Tables

**Table 1:** The c-miRNAs in BC papers.

Breast Cancer				
miRNA	Source	Population	Function	References
↑ miR-182; ↑ miR-375.	Serum	Pre- and post-menopausal BC patients and patients with benign tumors	Positive association with estrogen and progesterone receptors	[27]
↑ miR-331; ↓ miR-195.	Plasma/ Whole blood	Patients with metastatic BC compared to those suffering from local disease or those who are healthy subjects	Identification of metastatic disease	[28]
↑ miR-106a-3p; ↑ miR-106a-5p; ↑ miR-20b-5p; ↑ miR-92a-2-5p.	Plasma	BC patients and healthy controls	Upregulation in BC and prognostic significance	[26]
↑ miR-106a-5p; ↑ miR-19b-3p; ↑ miR-20b-5p; ↑ miR-92a-3p.	Serum	BC patients and healthy controls	Upregulation in BC and prognostic significance	[26]
↑ let-7b-5p; ↑ miR-106a-5p; ↑ miR-19a-3p; ↑ miR-19b-3p; ↑ miR-20a-5p; miR-223-3p; ↑ miR-25-3p; ↑ miR-425-5p; ↑ miR-451a; ↑ miR-92a-3p; ↑miR-93-5p; ↑ miR-16-5p.	Serum	BC patients and healthy controls	Distinguishing BC in different stages from controls	[29]
↕ miR-142-5p; ↕ miR-150-5p; ↕ miR-320a; ↕ miR-4433b-5p.	Serum	EVs derived for the sera of CT subjects, Luminal A BC patients and triple negative BC patients	Distinguishing BC patients from CT subjects	[30]
↑ miR-202; ↑ miR-21; ↑ miR-155; ↑ miR-23a; ↑ miR-130a; ↑ miR-145; ↑ miR-425-5p; ↑ miR-139-5p.	Plasma	BC patients from South Korea and Lebanese BC patients	Differential expression of circulating miRNAs in different ethnic groups	[31,32]
↑ miR-133a-3p; ↑ miR-497-5p; ↑ mir-24-3p; ↑ miR-125b-5p; ↓ miR-377-3p; ↓ miR-374c-5p; ↓ miR-324-5p; ↓miR-19b-3p.	Serum	Caucasian and Asian BC patients and healthy controls	Distinguishing between BC patients and healthy individuals	[33]
↑ miR-155.	Plasma	BC patients at diagnosis and after treatment	Potential role in diagnosis and therapeutic monitoring	[24]
↑ miR-21; ↑ miR-181a; ↑ miR-10b; ↓ miR-145; ↓ let-7a.	Plasma	Locally advanced BC patients at diagnosis, during treatment, and after tumor restriction	Differential expression of miRNAs in BC patients with respect to HCs. Expression levels of the miRNAs returned to control values once the treatment finished.	[34]
↑ miR-21; ↑ miR-55; ↑miR-10b; ↓ let-7a.	Plasma	Non-metastatic Luminal A patients undergoing the common treatments, such as surgery, chemotherapy, and radiotherapy	Treatments reversed the expression patterns of miRNAs	[35]
↑ Let-7a; ↓ miR-145.	Whole blood	Luminal B and HER2+ BCs	Predicting the response to NAC in BC patients	[36]
↑ miR-145.	Whole blood	Patients undergoing neoadjuvant chemotherapy across eight Irish centers.	Improved recurrence-free survival	[37]
↕ miR-718; ↕ miR-4516; ↕ miR-210; ↕ miR-125b-5p.	Plasma	Luminal B-HER2-negative patients undergoing NAC	Association with chemosensitivity in Luminal B-HER2-negative patients undergoing NAC	[38]
↕ miR-222, ↕ miR-20a; ↕ mir-451.	Plasma	HR+/HER2+ BC patients	Association with the chemosensitivity in a cohort of HR+/HER2+ BC patients	[39]
↑ miR-148a-3p; ↑miR-374a-5p.	Plasma	HER2-positive BC patients receiving trastuzumab-based neoadjuvant therapy (NeoALLTTO trial)	Prognostic significance in identifying patients likely to respond to therapy	[40]
↓ miR-200a; ↓ miR-200b; ↓ miR-141.	Plasma	BC patients receiving a complete cycle of systemic therapy	These values returned at the basal level upon the progression of disease, suggesting the potential effectiveness of these miRNAs in the management of metastatic BC	[43,44]
↑ miR-21; ↑ miR-23b; ↑ miR-200c; ↓mir-190.	Plasma	BC patients before adjuvant chemotherapy	Efficacy as biomarkers for BC recurrence	[41]
↑ miR-199a; ↓ miR-633b.	Plasma	Metastatic BC patients and healthy subjects	Correlation with chemoresistance in metastatic BC subjects	[45]

↑ = increased expression; ↓ = decreased expression; ↕ = differential expression.

**Table 2:** The c-miRNAs in LC papers.

Lung Cancer				
miRNA	Source	Population	Function	Reference
↓ miR-590-5p.	Plasma	80 NSCLC patients compared to healthy controls	Association between low levels of miR-590-5p and poor prognosis, in terms of median survival	[58]
↑ mir-2114; ↑mir-449c; ↑mir-2115.	Plasma	Lung adenocarcinoma and squamous cell carcinoma cases, compared with those from healthy individuals	Increased expression levels of mir-2114 and mir-449c in AC and mir-2115 in SCC; potential diagnostic significance	[59]
↕ miR-1285-3p; ↕ miR-205-5p; ↕ miR-1260a; ↕ miR-1260b; ↕ miR-3152-3p; ↕ miR-378b; ↕ miR-17-3p; ↕ miR-1202; ↕ miR-139-5p; ↕ miR-16-2-3p; ↕ miR-18a-3p; ↕ miR-23b-3p; ↕ miR-3907; ↕ miR-551b-3p; ↕ miR-93-3p.	Whole blood	LC patients (both NSCLC and SCLC), patients with non-tumor lung diseases, patients with no pulmonary diseases, and unaffected control participants.	miRNA signature was used to discriminate between LC-diagnosed subject and all other individuals	[60]
↕ let-7g-3p; ↕ miR-1202; ↕ miR-1285-3p; ↕ miR-17- 3p; ↕ miR-193a-5p; ↕ miR-205-5p; ↕ miR-21-3p; ↕ miR-3610; ↕ miR-4282; ↕ miR-4286; ↕ miR-452-3p; ↕ miR- 516a-3p; ↕ miR-572; ↕ miR-625-5p.			miRNA signature was used to discriminate between LC and non-tumor lung diseases	
↕ miR-1260a; ↕ miR-1260b; ↕ miR-1285-3p; ↕ miR-17-3p; ↕ miR-205-5p; ↕ miR-3152-3p; ↕ miR-374b-5p; ↕ miR-378b; ↕miR-564.			miRNA signature was used to discriminate between early-stage LC patients vs. individuals without LC	
↑ miR-21; ↓ miR-638; ↓ miR148; ↓ miR-152.	Whole blood	NSCLC patients and non-cancerous subjects	Diagnostic efficacy in distinguishing between LC patients and non-cancerous subjects	[61]
↓ let-7a-5p; ↓ miR375; ↑ miR-1-3p; ↑miR-1291; ↑ miR-241-3p.	Serum	NSCLC patients and matched controls, including smokers and nonsmokers, male and female	miRNA signature was used for the identification of early-stage NSCLC	[62]
↑ miR-191.	Serum	NSCLC patients and controls	Upregulation of miRNA in cancerous vs. non-cancerous tissues and its role in sustaining the proliferation and migration of LC cells in hypoxic conditions	[63,64]
↕ let-7-5p; ↕ miR-184; ↕ miR-22-3p.	Plasma	NSCLC patients and high-risk subjects	Let-7-5p, miR-184 from Evs, and miR-22-3p from c-miRNAs were able to discriminate between the two groups.	[65]
↑ miR-378.	Serum	NSCLC patients, subjects with a non-malignant disease, and healthy controls	The expression levels of exosomal miR-378 increased in cancerous sera compared to heathy sera; this increase was also associated with lymph node metastasis and the TNM stage	[66]
↓ miR-200c-3p; ↓ miR-21-5p; ↓ miR-28-5p.	Plasma	Small cohort of advanced NSCLC patients treated with a single-agent anti–PD-1 or an anti–PD-L1 antibody	Expression levels of miRNAs significantly decreased in responders when compared to non-responder NSCLC patients	[69]
↑ miR-93; ↑ miR-138-5p; ↑ miR-200; ↑ miR-27a; ↑ miR-424; ↑ miR-34a; ↑ miR-28; ↑ miR-106b; ↑ miR-193a-3p; ↑ miR-181a.	Serum	NSCLC patients undergoing immunotherapy and divided into responder and non-responder subjects	10-fold increase in the expression levels of miRNAs from pre- to post-treatment; the highly expressed signature was further associated with the improvement of progression-free survival	[70]
↕ miR-215-5p; ↕ miR-411-3p; ↕ miR- 493-5p; ↕ miR-494-3p; ↕ miR-495-3p; ↕ miR-548j-5p; ↕ miR-93-3p.	Serum	NSCLC patients treated with nivolumab	Association with OS after treatment with the immune check point inhibitor nivolumab	[71]
↑ miR-202.	Plasma	NSCLC patients treated with first-line platinum-based chemotherapy.	Correlation with disease progression in NCSLC patients and a prognostic significance for shorter progression-free survival and OS	[72]

↑ = increased expression; ↓ = decreased expression; ↕ = differential expression.
